# The Punicalagin Metabolites Ellagic Acid and Urolithin A Exert Different Strengthening and Anti-Inflammatory Effects on Tight Junction-Mediated Intestinal Barrier Function *In Vitro*

**DOI:** 10.3389/fphar.2021.610164

**Published:** 2021-03-10

**Authors:** Nina A. Hering, Julia Luettig, Britta Jebautzke, Jörg D. Schulzke, Rita Rosenthal

**Affiliations:** ^1^ Department of General and Visceral Surgery, Charité – Universitätsmedizin Berlin, Berlin, Germasny; ^2^ Institute of Clinical Physiology/Nutritional Medicine, Charité – Universitätsmedizin Berlin, Berlin, Germany

**Keywords:** barrier function, ellagic acid, punicalagin, tight junction, urolithin A

## Abstract

**Scope:** Ellagitannins are polyphenols found in numerous fruits, nuts and seeds. The elagitannin punicalagin and its bioactive metabolites ellagic acid and urolithins are discussed to comprise a high potential for therapeutically or preventive medical application such as in intestinal diseases. The present study characterizes effects of punicalagin, ellagic acid and urolithin A on intestinal barrier function in the absence or presence of the proinflammatory cytokine tumor necrosis factor-α (TNFα).

**Methods and Results:** Transepithelial resistance (TER), fluorescein and ion permeability, tight junction protein expression and signalling pathways were examined in Caco-2 and HT-29/B6 intestinal epithelial cell models. Punicalagin had less or no effects on barrier function in both cell models. Ellagic acid was most effective in ileum-like Caco-2 cells, where it increased TER and reduced fluorescein and sodium permeabilities. This was paralleled by myosin light chain kinase two mediated expression down-regulation of claudin-4, -7 and -15. Urolithin A impeded the TNFα-induced barrier loss by inhibition of claudin-1 and -2 protein expression upregulation and claudin-1 delocalization in HT-29/B6.

**Conclusion:** Ellagic acid and urolithin A affect intestinal barrier function in distinct ways. Ellagic acid acts preventive by strengthening the barrier per se, while urolithin A protects against inflammation-induced barrier dysfunction.

## Introduction

Health benefits of the ellagitannin punicalagin and its metabolites are being extensively discussed in recent years. Punicalagin can be found in pomegranate (*Punica granatum*), raspberries (*Rubus idaeus*), strawberries (*Fragaria sp.*) or walnuts (*Juglans regia*). Especially pomegranate has already been applied for thousands of years in the traditional medicine (Longtin, 2003) to benefit from its anti-diarrhea (Das et al., 1999; Zhao et al., 2018), anti-oxidant, anti-obesity, anti-cancerogenic, and anti-inflammatory properties (reviewed in Saeed et al., (2018). Ellagitannins are not directly absorbed into the blood stream (Seeram et al., 2004) but hydrolyzed. In the stomach and intestine, hydrolysis of punicalagin yields ellagic acid (EA), which in turn is metabolized to urolithin A and B by the intestinal microbiota (Cerdá et al., 2005; Espín et al., 2007). Several studies showed these metabolites exert anti-inflammatory effects in the intestine. In a mouse model of ulcerative colitis, ellagic acid was effective in reducing intestinal inflammation by inhibiting cyclooxygenase-2 and inducible nitric oxide synthases. It impeded pro-inflammatory signaling via nuclear factor 'kappa-light-chain-enhancer' of activated B-cells (NFκB) and signal transducers and activator of transcription 3 (STAT3) (Marín et al., 2013). A very recent cell culture study reported ellagic acid to inhibit pro-inflammatory effects of tumor necrosis factor α (TNFα). This involved loss of epithelia barrier function, upregulation of interleukin-6 and -8 secretion and induction of oxidative stress by impeding TNFα-induced signaling via NF-κB, extracellular signal-regulated kinases 1/2 (ERK1/2) and myosin light chain kinase (MLCK) (Iglesias et al., 2020). Urolithin A (UroA) can be detected at relatively high amounts in the colon. Its anti-inflammatory activities were reported from a rat model of colitis (Larrosa et al., 2010) and a colon fibroblast model (Giménez-Bastida et al., 2012).

Intestinal inflammation causes intestinal barrier dysfunction [(reviewed in Hering et al., (2012)). The intestinal barrier integrity plays a central role for gut health by preventing an abandoned passage of antigens, allergens, bacterial toxins or other noxious agents from the intestinal lumen into the mucosa and blood circulation. The intestinal epithelium is built up by a single row of epithelial cells, which are connected by tight junctions (TJs) at their most apical point. Dependent on the physiological condition, the epithelial TJs regulates the paracellular passive passage of water and nutrients. This is achieved by the specific interplay of different TJ proteins, including the large family of claudins, TJ-associated MARVEL proteins (Mineta et al., 2011), such as tricellulin, and junctional adhesion molecules (Raleigh et al., 2010). These transmembrane proteins are connected to intracellular scaffold proteins (e.g. zonula occludens proteins, ZO−1-3) and form a meshwork of numerous horizontally oriented strands surrounding the epithelial cells. Changes to this defined composition can result in altered barrier function. Pro-inflammatory cytokines, as e.g. tumor necrosis factor α (TNFα) are well-known to cause barrier dysfunction by inducing epithelial apoptosis and by affecting TJ architecture, including claudin protein expression and delocalization (Hering and Schulzke, 2009).

So far, little is known about the impact of punicalagin, ellagic acid or urolithin A on intestinal barrier function. Objective of the present study was to elucidate their putative protective and barrier strengthening properties on epithelial TJ integrity per se or in state of inflammation. As their bioavailability differs along the gastrointestinal tract (Espín et al., 2013), we hypothesized that these bioactive compounds might act distinctly on barrier function in ileum or colon. Therefore, we investigated two different intestinal cell lines, ileum-like Caco-2 cells and HT-29/B6 colon cells.

## Materials and Methods

### Cell Culture and Dosage Information

Caco-2 cells are epithelial cells derived from a colorectal adenocarcinoma (ATCC® HTB-37™). However, under specific culturing conditions Caco-2 cells differentiate and polarize such that they functionally and morphologically resemble the phenotype of distal ileum enterocytes. They are characterized by absorptive capabilities and active transport pathways, possess enzymatic activities and an apical brush border. When cultured on filter supports Caco-2 cells grow as polarized monolayers with epithelial TJs (Hidalgo et al., 1989).

In the present study Caco-2 cells were cultured using Minimum Essential Medium Eagle AqmediaTM containing 15% bovine serum and 1% penicillin/streptomycin (all Sigma-Aldrich, Schnelldorf, Germany). Within two weeks after seeding on permeable Millicell PCF filters (0.6 cm^2^ effective area; 0.4 μm pores, Millicell PCF, Millipore, Schwalbach, Germany), they grew to confluence and transepithelial resistance (TER) was usually ranging between 280 and 450 Ω⋅cm^2^. Fifteen days old monolayers were challenged with different doses of punicalagin (10, 25, 50, 150 and 250 μM; Sigma Aldrich), ellagic acid hydrate (50, 100, 150, 200 and 300 μM; Sigma-Aldrich) or urolithin A (25, 50, 100, 150 and 250 μM; Sigma-Aldrich). Substances were dissolved in dimethyl sulfoxide (DMSO). To avoid osmotic effects the monolayers were usually challenged from the apical and basolateral side. Control monolayers were treated with equal amounts of DMSO. Three aspects considered the optimal doses. 1) TER effect, 2) reproducibility (demonstrated by SEM), and 3) the amount of dimethyl sulfoxide (DMSO). Due to the toxicity of DMSO, concentrations above 1% DMSO were not considered for optimal doses.

Myosin light chain kinase inhibitor PIK (150 µM) was pre-incubated on Caco-2 monolayers 2 h before challenging with EA. Phosphorylation events were studied under serum free conditions.

The colon carcinoma cell line HT-29/B6 is a subclone of the human colon carcinoma cell line HT-29 (Kreusel et al., 1991) and was cultured on permeable filter supports (0.6 cm^2^ effective area; 3.0 μm pores, Millicell PCF, Millipore) using RPMI medium (Sigma-Aldrich) containing 10% bovine serum and 1% penicillin/streptomycin. Monolayers grew confluent within one week, giving a TER of at least 350 Ω⋅cm^2^and were pre-incubated with 10 µM punicalagin, 150 µM ellagic acid and 150 µM or 250 µM urolithin A from both sides. Two hours later 500 U/ml TNFα (Pepro Tech, Hamburg, Germany) were added to the basal compartment.

Changes in barrier integrity were assessed by measuring transepithelial resistance (TER) with a pair of chopstick electrodes at 37 C as described before (Heller et al., 2005).

### Permeability Measurements

For permeability measurements, monolayers were mounted into Ussing chambers. The standard bathing solution contained: 140 mM Na^+^, 123.8 mM Cl^−^, 5.4 mM K^+^, 1.2 mM Ca^2+^, 1.2 mM Mg^2+^, 2.4 mM HPO_4_^2−^, 0.6 mM H_2_PO_4_^−^, 21 mM HCO_3_^−^ and 10 mM D (+) -glucose. Flux measurements were performed under voltage-clamp conditions with 0.1 mM fluorescein (332 Da, Sigma-Aldrich, Schnelldorf, Germany), which was added to the apical side of the monolayer. Samples were collected from the basolateral side at defined time points. Fluorescence was measured in a spectrofluorimeter (Infinite M200, Tecan, Männedorf, Austria) at 525 nm. Fluorescein permeability *p* (cm⋅s^−1^) was calculated from the ratio of flux J (mol⋅h^−1^⋅cm^−2^) over concentration Δc (mol/L): *p* = J/Δc.

For the measurement of Na^+^ and Cl^−^permeability, voltage and TER were monitored while reducing NaCl concentration in one hemichamber. This was done by switching to a solution containing a reduced concentration of NaCl and mannitol for balancing osmolality. All other components were equivalent to standard solution. NaCl permeability was determined from dilution potentials and the Goldmann–Hodgkin–Katz equation as previously reported (Amasheh et al., 2002; Günzel et al., 2009; Yu et al., 2009).

### Western Blot Analyses

For expression analyses, proteins were extracted using ice-cold lysis buffer, including 10 mM Tris, pH 7.5, 150 mM NaCl, 0.5% Triton X-100, 0.1% SDS and complete protease inhibitor mixture (Roche, Basel, Switzerland). For phosphorylation studies, the lysis buffer contained 20 mM Tris (pH 7.5), 150 mM NaCl, 1 mM EDTA, 1 mM EGTA, 1% Triton X-100, 2.5 mM Na_2_H_2_P_2_O_7_, 1 mM β-glycerolphosphate, 1 mM Na_3_VO_4_, 1 mg ml^−1^ leupeptin, 1 mM PMSF and complete protease inhibitor. For analysis of caspase-3 cleavage, cell lysis was performed as described recently (Hering et al., 2017).

Protein extracts (15–40 mg) were separated by SDS-gel electrophoresis and blotted on PVDF membrane. Antibodies used for immunodetection: anti-claudin-1 to −4, claudin−7 and −15 (1:1000, Thermo Fisher Scientific, Bremen, Germany), β-actin (1:5000, Sigma-Aldrich), anti-phospho-myosin light chain 2, anti-myosin light chain 2 (Cell Signaling Technology, Danvers, MA, USA), and anti-caspase 3 (1:1000; Cell Signaling Technology). Chemiluminescent imaging of bound antibodies was performed with peroxidase-conjugated goat anti-rabbit IgG or goat anti-mouse IgG antibodies, chemiluminescence substrate Lumi-LightPLUS (Roche) and the FX7detection system (Vilber Lourmat, Eberhardzell, Germany). Densitometry was carried out with Image Studio Light (LI-COR Biosciences; NE, United States) and values were normalized to β-actin that served as internal loading control.

### Immunofluorescence Staining and Confocal Laser Scanning Microscopy

Monolayers were rinsed with PBS and fixed with 4% of paraformaldehyde for 10 min at room temperature. After permeabilization with 0.5% Triton X-100 monolayers were blocked with 5% goat serum and 1% bovine serum albumin. Immunostaining was carried out with primary antibodies for anti-ZO-1 (1:100), anti-claudin-1, -4, -7 and -15 (all 1:100; Thermo Fisher Scientific) at 4°C over night. Counterstaining was performed using Alexa Fluor 488 goat anti-mouse and Alexa Fluor 594 goat anti-rabbit IgG (1:1000; Thermo Fisher Scientific) as described before (Luettig et al., 2016). Nuclei were stained with 4′,6-Diamidin-2-phenylindol (DAPI) (1:5000). Intensity and localization of claudins was analyzed by confocal laser scanning microscopy (LSM 780, Zeiss, Jena, Germany).

### Freeze Fracture Electron Microscopy

Freeze fracture electron microscopy analysis was performed and quantified as described elsewhere in detail (Krug et al., 2009).

### Statistical Analyses

Statistical analysis was done using Student’s t-test and Bonferroni-Holm adjustment in case of multiple comparison. All data are expressed as mean ± SEM. *p* < 0.05 was considered significant (*p < 0.05; **p < 0.01; ***p < 0.001 or ^#^ *p* < 0.05; ^##^ *p* < 0.01; ^###^ *p* < 0.001).

## Results

### Stabilizing Effect of Punicalagin, Urolithin A and Ellagic Acid on Barrier Properties of Caco-2 Intestinal Cells

**FIGURE 1:**
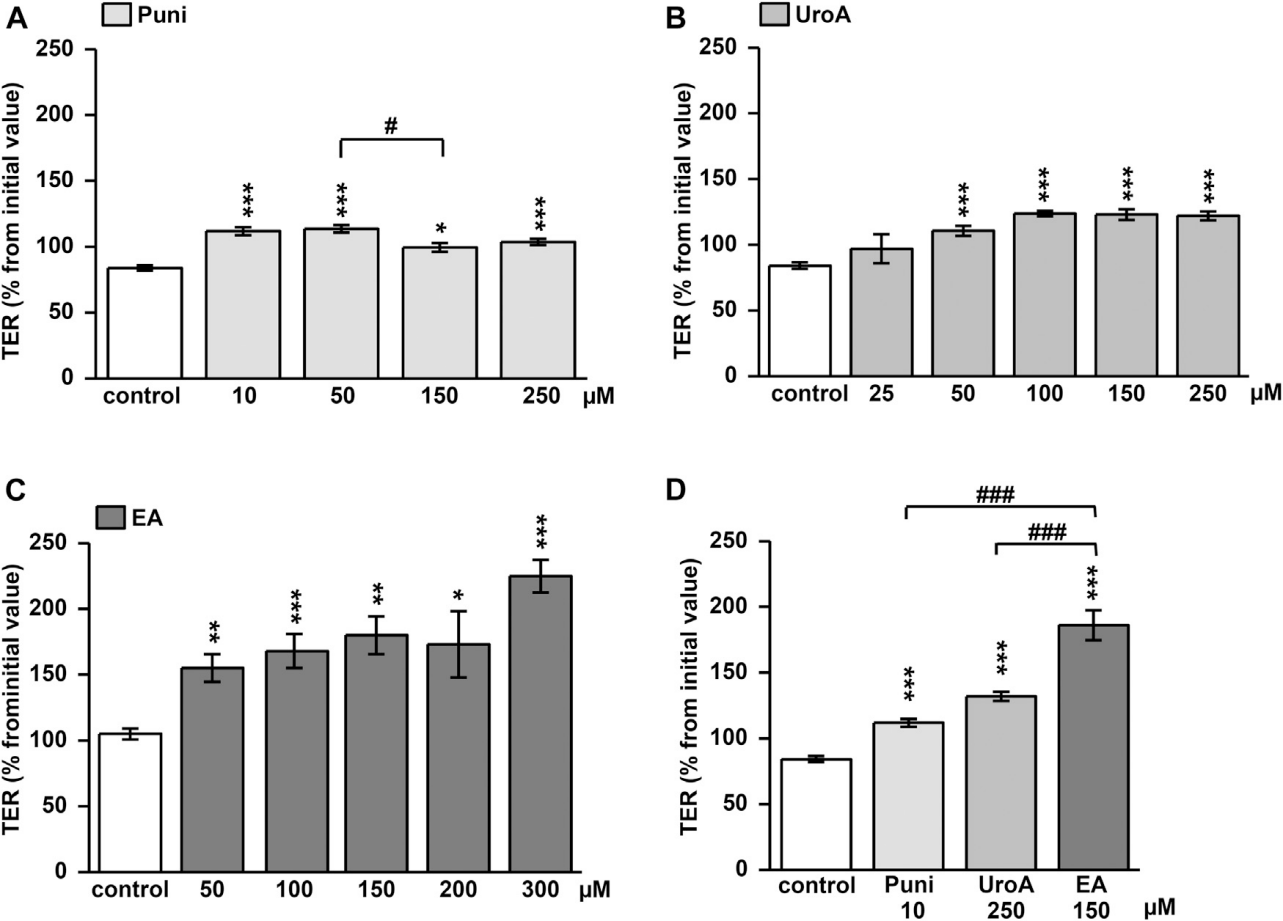
Impact of punicalagin, ellagic acid and urolithin A on transepithelial resistance (TER) in Caco-2 monolayers. Changes in TER from initial values were measured at 24 h after challenging monolayers with different doses of **(A)** punicallagin (Puni) (p*<0.05, ***p < 0.001 vs. control; ^#^ *p* < 0.05 50 vs. 150 μM; *n* = 3–12) **(B)** urolithin A (UroA) (***p < 0.001 vs. control; *n* = 3–12) or **(C)** ellagic acid (EA) (p*<0.05, **p < 0.01, ***p < 0.001 vs. control; *n* = 6–15) **(D)** Comparison of the most effective dose of each compound on TER at 24 h after challenging (***p < 0.001 vs. control;^###^ *p* < 0.001 vs. ellagic acid; *n* = 6–9).

**FIGURE 2:**
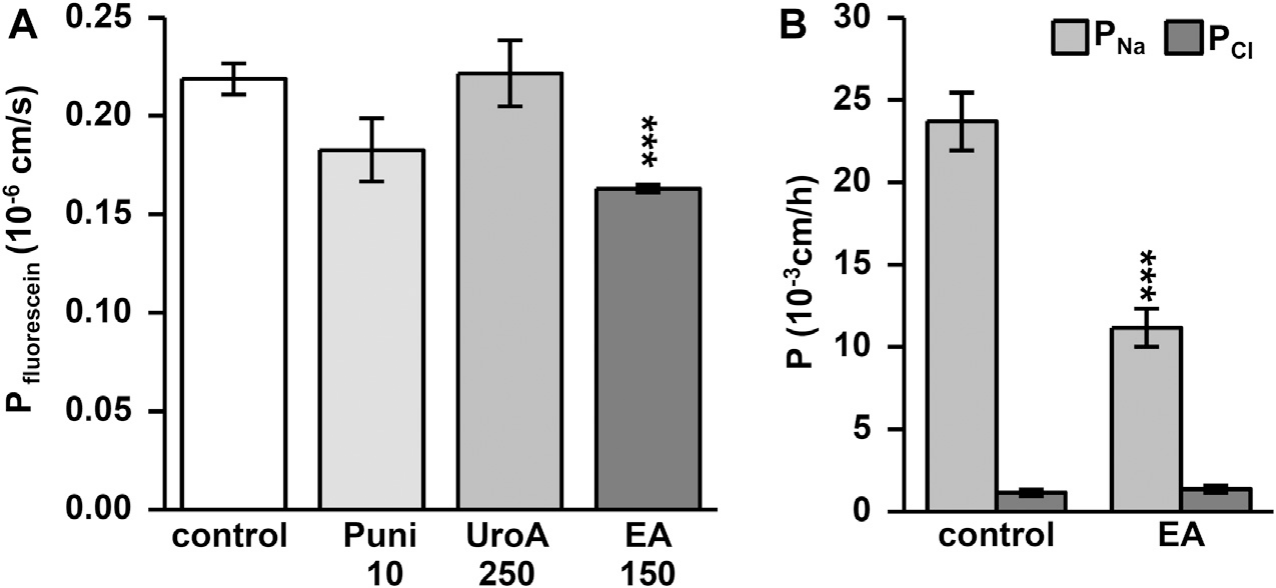
Ellagic acid decreases permeability to fluorescein and sodium in Caco-2 monolayers. Permeability measurements were performed in Ussing chambers at 24 h after challenging. Ellagic acid (EA) reduces permeability to the **(A)** 332 Da marker molecule fluorescein (***p < 0.001 vs. control; *n* = 7–9) and **(B)** to sodium ions (***p < 0.001 vs. control; *n* = 8) compared to untreated controls.

Punicalagin increased TER slightly from initial values in Caco-2 monolayers within 24h (Figure 1A; *p* < 0.05, *p* < 0.001 vs. control). This TER increase could not be enhanced dose-dependently (Figure 1A; *p* < 0.05 Puni 50 vs. 150 µM). In contrast, the TER increase induced by urolithin A was more pronounced (Figure 1B). While 25 µM was not effective, doses up to 100 µM UroA increased TER significantly from control (Figure 1B; *p* < 0.001 vs. control). Higher doses than 100 µM of urolithin A had no further increasing effects (Figure 1B). Ellagic acid caused the strongest TER increase within 24 h. 50 µM was as effective as 200 μM EA compared to control (Figure 1C; *p* < 0.001 and *p* < 0.05 vs. control). Comparing the most effective dose of each compound in one experiment proved ellagic acid (150 µM) to induce the strongest TER increase in Caco-2 monolayers, followed by urolithin A (250 µM) and punicalagin (10 µM) (Figure 1D; *p* < 0.001 vs. control and *p* < 0.001 vs. EA). The TER increase induced by 150 µM ellagic acid was paralleled by a permeability decrease of the 332Da marker molecule fluorescein in Caco-2 monolayers (Figure 2A; *p* < 0.001 vs. control). Neither 250 µM urolithin A nor 10 µM punicalagin reduced fluorescein permeability (Figure 2A). Measurements of dilution potentials for sodium and chloride showed that ellagic acid restricted sodium permeability, but not chloride permeability in Caco-2 monolayers (Figure 2B; *p* < 0.001 vs. control). The permeability ratios of sodium and chloride (P_Na_/P_Cl_) were reduced 3-fold from 28 ± 7 in control to 9 ± 1 in monolayers challenged with ellagic acid (*p* < 0.05 vs. control).

**FIGURE 3:**
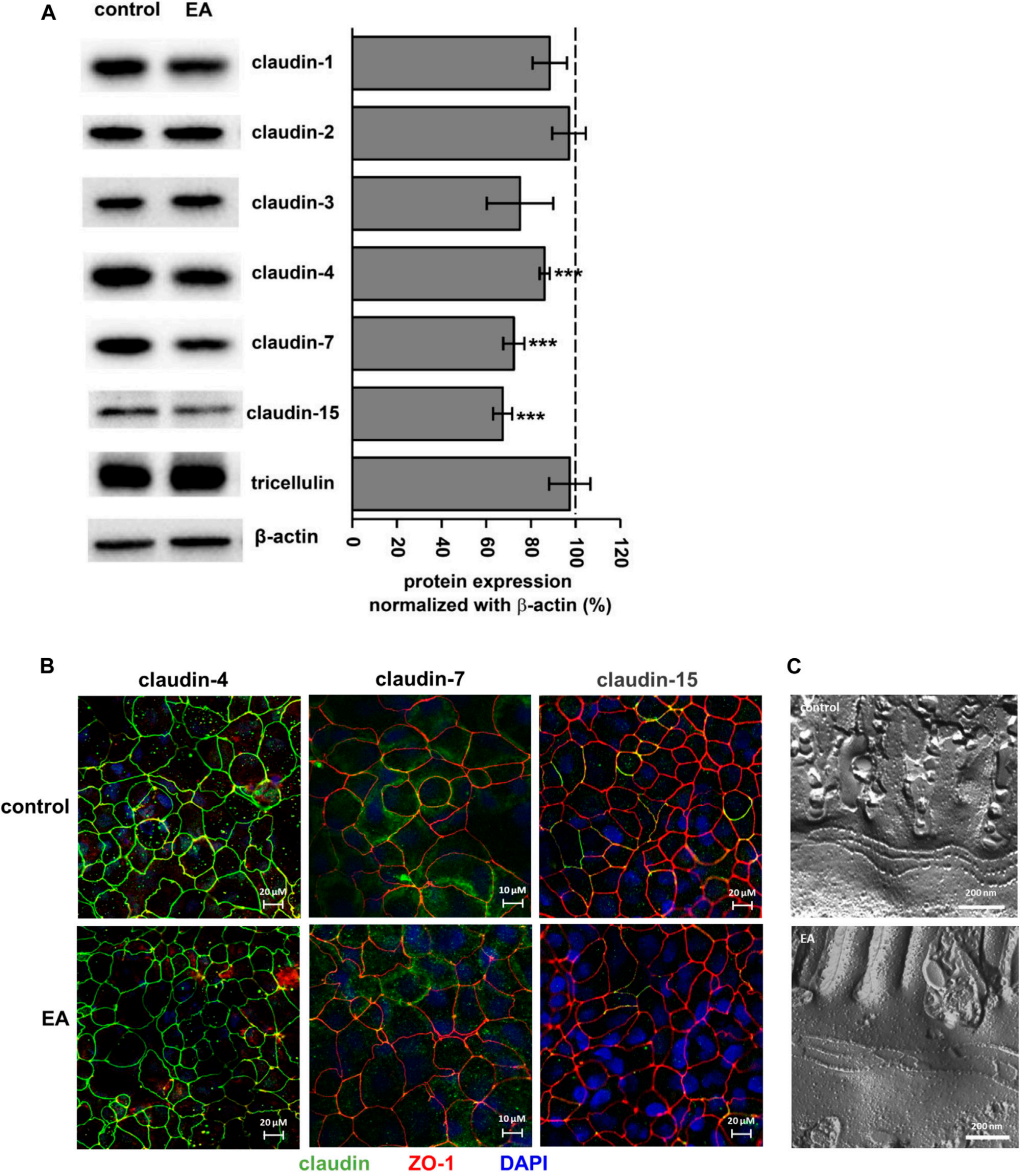
Ellagic acid reduces protein expression of TJ proteins claudin-4, -7 and -15 in Caco-2 monolayers **(A)** Representative Western blots and densitometry show reduced expression of claudin-4, -7 and -15 in ellagic acid (EA)-challenged monolayers (p***<0.001 vs. control; *n* = 7–10) **(B)** Representative micrographs of immunofluorescence staining show claudin-4, claudin-7 and claudin-15 in control and elllagic acid-challenged monolayers (green). ZO-1 served as TJ marker (red), nuclei were DAPI stained (blue) (*n* = 3 each) **(C)** Representative freeze-fracture EM micrographs of control and ellagic acid-challenged monolayers show no differences in TJ ultra-structure.

To analyze the barrier effect of ellagic acid in more detail, TJ protein expression was examined after 24 h of challenging monolayers with 150 µM ellagic acid. This reduced the protein level of claudin-4, -7 and -15 (Figure 3A; *p* < 0.0001 vs. control), but did not affect claudin-1, -2 or -3 and tricellulin (Figure 3A). Claudin-5 and -8 were not expressed in our Caco-2 cells. Expression down regulation of claudin-4, -7 and -15 obtained from Western blot analyses were confirmed by confocal laser scanning microscopy of immunostained Caco-2 monolayers. The intensity of claudin-4, claudin-7 and claudin-15 signals was reduced in monolayers challenged with 150 µM ellagic acid (Figure 3B). Especially claudin-7 and -15 were only present in single cells of the Caco-2 control monolayers. Ellagic acid treatment reduced the number and frequency of these claudin-7 or claudin-15 positive cells (Figure 3B). Overall TJ ultrastructure was not influenced by ellagic acid. Morphometric analyses of freeze fracture electron micrographs revealed no alterations in TJ ultra-structure (Figure 3C). TJ strand number (3.4 ± 0.2 vs. 3.2 ± 0.1 in control), density (23 ± 3 vs. 22 ± 2 in control) and type, meshwork depth (147 ± 17 vs. 143 ± 9 in control), and number of strand breaks did not differ from control in ellagic acid-challenged Caco-2 monolayers.

**FIGURE 4:**
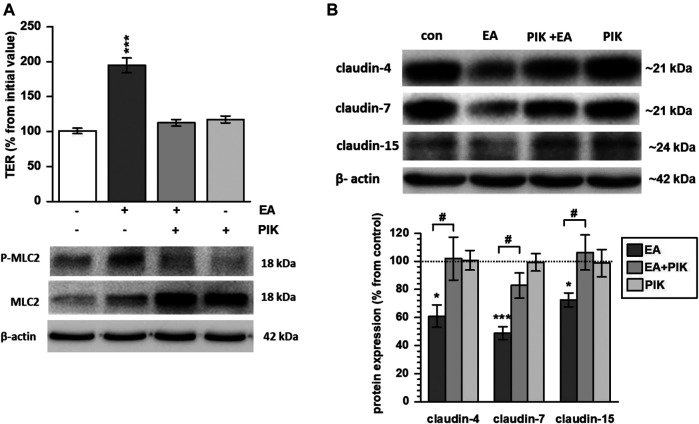
Ellagic acid strengthens barrier function via MLC2 signaling in Caco-2 monolayers **(A)** Pre-incubation with the MLCK specific inhibitor PIK impeded ellagic acid (EA)-stimulated MLC2 phosphorylation and TER increase in Caco-2 monolayers at 24 h after challenge (p***<0.001 vs. control; *n* = 9)., **(B)** Representative Western blots and quantification show that PIK inhibition blocked ellagic acid-stimulated down regulation of claudin-4, -7 and -15 in parallel (*p < 0.05; ***p < 0.001 vs. control; ^#^ *p* < 0.05 ellagic acid vs. ellagic acid + PIK; *n* = 6–9).

Signaling was studied by assessing different kinase inhibitors. Although ellagic acid induced phosphorylation of p38 and STAT3, specific phosphorylation inhibition of these kinases could not impede the ellagic acid-stimulated TER increase (data not shown). In contrast, inhibition of MLCK by PIK prevented Myosin Light Chain 2 (MLC2) phosphorylation and blocked the ellagic acid-induced TER increase (Figure 4A; *p* < 0.001 vs. control). In parallel, PIK impeded the ellagic acid-depended expression down-regulation of claudin-4, -7 and -15 (Figure 4B; *p* < 0.05 PIK + ellagic acid vs. ellagic acid alone). In PIK + ellagic acid co-treated monolayers, protein levels did not differ from control (Figure 4B).

### Protective Effect of Urolithin A on TNFα-Induced Barrier Loss in HT-29/B6 Cells

**FIGURE 5:**
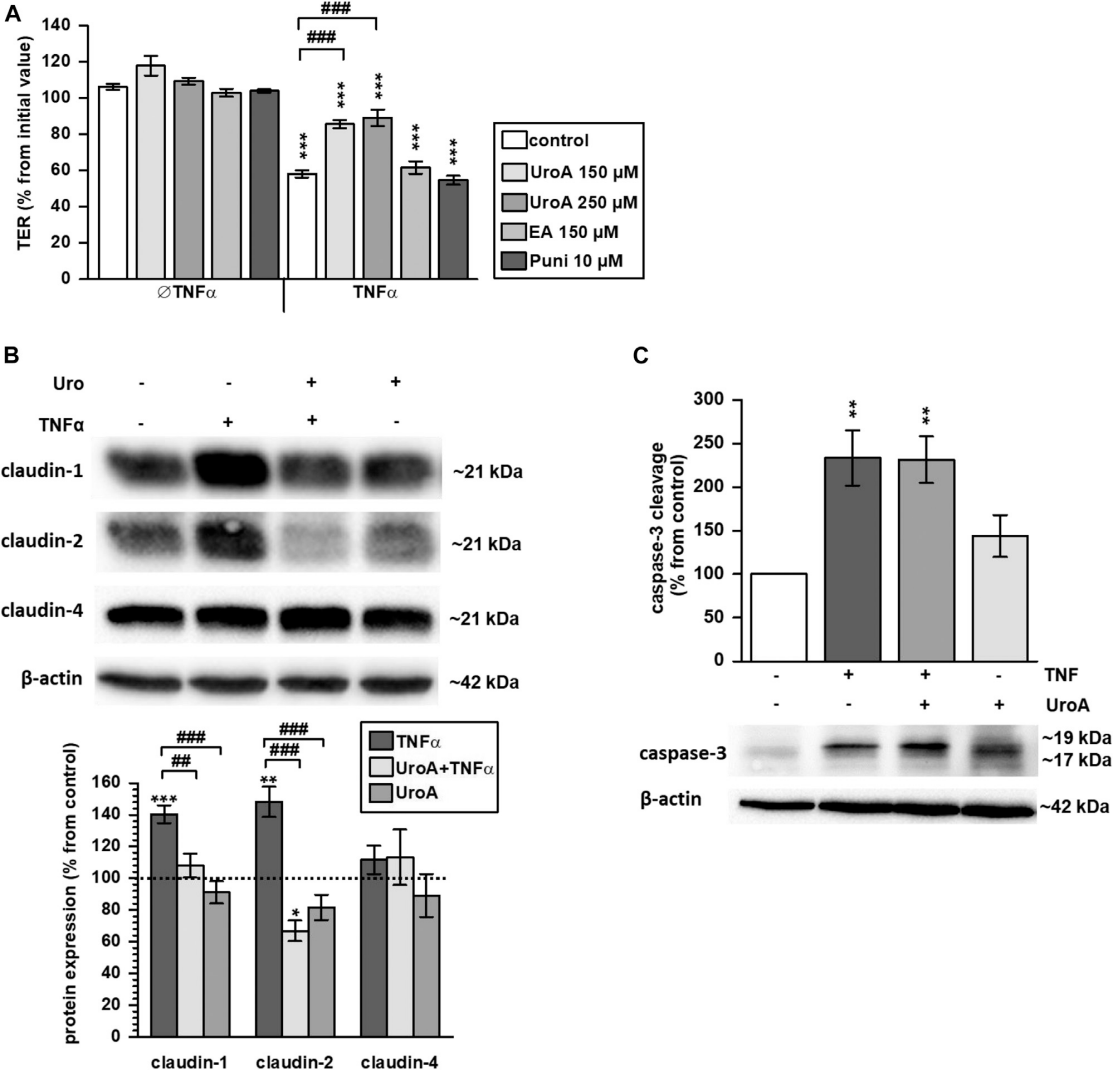
Urolithin A antagonizes the TNFα–induced TER decrease and expression changes to claudin-1 and two in HT-29/B6 monolayers **(A)** 150 and 250 µM urolithin A (UroA) partially prevented the TNFα-caused TER decrease in HT-29/B6 monolayers (***p < 0.001 vs. control; ^###^ *p* < 0.001 TNFα vs. urolithin A + TNFα; *n* = 6–12) **(B)** 150 µM urolithin A impeded TNFα-induced up-regulation of claudin-1 and -2 completely, as shown by representative Western blots and densitometry (*p < 0.05, **p < 0.01, ***p < 0.001 vs. control; ^##^ *p* < 0.01, ^###^ *p* < 0.001 TNFα vs. urolithin A + TNFα or urolithin A; *n* = 5–8) **(C)** but 150 µM urolithin A did not prevent caspases-3 cleavage caused by TNFα (**p < 0.01 vs. control; *n* = 5–6).

HT-29/B6 monolayers were challenged with the pro-inflammatory cytokine TNFα that caused a TER drop of about 40% within 24 h (Figure 5A; *p* < 0.001 vs. control). Pretreatment with 150 µM or 250 µM urolithin A partially reversed this TNFα-induced decrease (*p* < 0.001 vs. TNFα), while both urolithin A doses were comparably effective (Figure 5A). In contrast, 10 µM punicalagin or 150 µM ellagic acid did not inhibit the TNFα-induced TER decrease (Figure 5A). TNFα is well known to upregulate expression of claudin-1 and -2 in HT-29/B6 cells. Within the present study, pre-incubation with 150 µM urolithin A prevented the TNFα-stimulated up-regulation of both claudins. Western blotting showed an increase of about 40% in claudin-1 expression by TNFα (Figure 5B; *p* < 0.001 vs. control), while in urolithin A co-treated monolayers expression remained at the control level (Figure 5B). Claudin-2 protein level was increased by TNFα about 50% from control values (Figure 5B; *p* < 0.01 vs. control). Pre-treatment with urolithin A reduced claudin-2 expression to 67% from control (Figure 5B; *p* < 0.05 vs. control). Claudin-4 expression was not affected by TNFα or urolithin A (Figure 5B).

**FIGURE 6:**
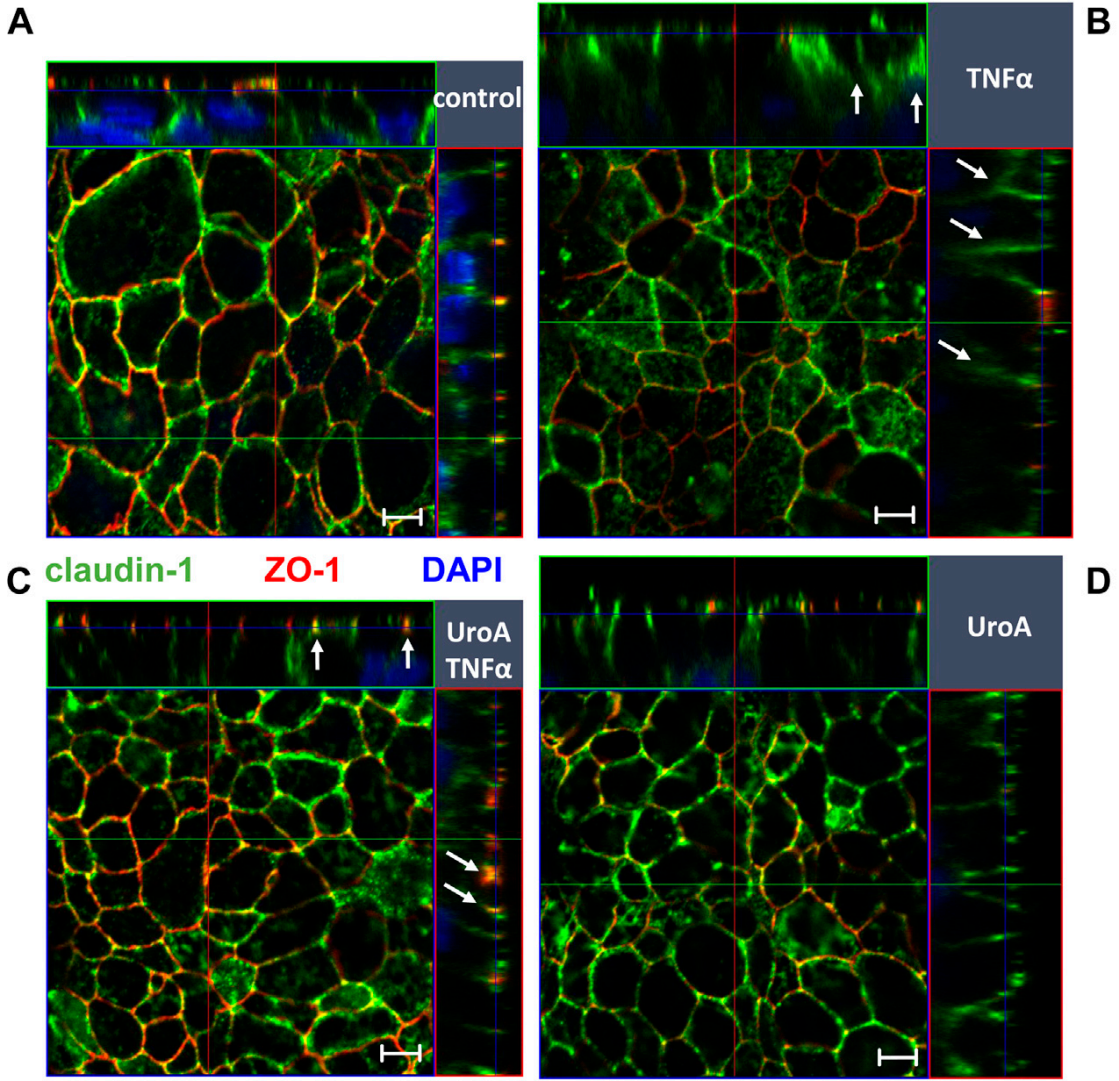
Effect of Urolithin A on TNFα-caused claudin-1 delocalization in HT-29/B6 monolayers. Localization of claudin-1 was studied by confocal laser scanning microscopy in **(A)** control **(B)** TNFα **(C)** urolithin A (UroA) +TNFα and **(D)** urolithin A-challenged monolayers. Merging of claudin-1 (green) with the TJ marker protein ZO-1 (red) was assessed by z-stack imaging, nuclei are DAPI stained (blue). TNFα caused delocalization of claudin-1 (indicated by white arrows in **(B)**), while parallel Urolithin A treatment enhanced claudin-1-ZO-1 merging (indicated by white arrows in **(C)**). Bars indicate 5 µm.

Because TNFα causes epithelial apoptosis in HT-29/B6 cells, effects of urolithin A on caspase-3 cleavage were examined by Western blotting. As expected, TNFα enhanced caspase-3 cleavage compared to untreated controls (Figure 5C; *p* < 0.01 vs. control). Urolithin A did not reduce TNFα-induced caspase-3 cleavage, but seemed to stimulate it. However, this did not reach statistical significance (Figure 5C). Beside expression regulation, TNFα caused claudin-1 redistribution out off the TJ into subapical compartments compared to untreated controls (Figures 6A,B, indicated by white arrows in Figure 6B). Representative micrographs of immunofluorescence staining showed increased merging of claudin-1 with the TJ marker protein ZO-1 in monolayers that were co-challenged with TNFα and urolithin A (indicated by white arrows in Figure 6C). In urolithin A-challenged monolayers, claudin-1 intensity and localization appeared not different from control (Figure 6D).

## Discussion

Epithelial barrier function is a key feature of intestinal health. The present study examined the molecular mechanisms behind the health claims of the bioactive polyphenol punicalagin and its metabolites ellagic acid and urolithin A on epithelial barrier function *in vitro*. Our data revealed that especially ellagic acid and urolithin A affect barrier function in different ways in our two cell models. Ellagic acid strengthened barrier function per se by reducing the expression of pore-forming claudin-4, -7 and -15 via MLC2 signaling in ileum-like Caco-2 cells. Urolithin A impeded the pro-inflammatory dysregulation and/or redistribution of claudin-2 and -1 in colonic HT-29/B6 cells. Punicalagin alone had only little or no effects on barrier function in the two cell models.

The constitution of the epithelial TJ, particularly its composition of pore-forming and sealing TJ proteins, is crucial for intestinal barrier function. Barrier integrity can be estimated by measuring TER and paracellular permeability of differently sized molecules or ions. Especially in Caco-2 cells, ellagic acid turned out to strengthen epithelial barrier function per se, displayed by a strong increase in TER, reduction in fluorescein and sodium permeability and down regulation of claudin-4, -7 and -15 expression.

Tight junction ultra-structure was not changed as demonstrated by freeze fracture electron microscopy. Subsequently, barrier effects are not due to changes in overall protein content but to TJ protein composition. Especially, the downregulation of channel forming claudins seems rational. Claudin-15 is predominantly expressed in the small intestine (Inai et al., 2005; Fujita et al., 2006; Holmes et al., 2006) and forms a paracellular cation‐ (Colegio et al., 2002; Van Itallie et al., 2003; Samanta et al., 2018) and water-selective channel (Rosenthal et al., 2020). Overexpression of claudin-15 in Caco-2 cells caused a decrease in TER (Takehara et al., 2009). The physiological function of claudin-4 and -7 is less clear as it is not consistent and seems to depend on the interactions with other TJ proteins and differs in different cell types and conditions (Günzel and Fromm, 2012). Two studies on flavonoids in Caco-2 cells reported quercetin (Amasheh et al., 2008) or kaempferol (Suzuki et al., 2011) to enhance barrier function by up-regulating claudin-4 expression. In contrast, in a cell culture study on kidney cells, claudin-4 was suggested to act as a chloride channel. Knock down of claudin-4 resulted here in a decrease in P_Na_/P_Cl_, which was paralleled by an TER increase (Hou et al., 2010). In an epithelial co-culture model of Caco and HT29-MTX cells, a decrease in TER induced by oxidative stress was associated with an increase in claudin-7 (Bianchi et al., 2019). In kidney cells, claudin-7 overexpression was associated with a decrease in P_Cl_ and an increase in P_Na_ (Alexandre et al., 2005). Taken together, it seems rational that especially the reduction of claudin-15 and claudin-7 are responsible for the decrease in sodium permeability and the TER increase in ellagic acid-challenged Caco-2 monolayers.

Ellagic acid enhanced the phosphorylation of STAT3, p38 and MLC2 in Caco-2 cells. However, only inhibition of MLC2 phosphorylation by the MLCK-inhibitor PIK impeded the ellagic acid-stimulated TER increase, suggesting a central role of MLC2 in ellagic acid-dependent barrier regulation. Phosphorylation of the MLC2 by MLCK is linked to actomyosin contraction and TJ regulation (Turner et al., 1999; Zolotarevsky et al., 2002; Shen et al., 2006). We showed that phosphorylation inhibition of MLC2 impeded the ellagic acid-induced TER increase and down-regulation of claudin-4, -7 and -15. So far, the role of non-muscle MLCK and MLC2 for barrier function was predominantly discussed with respect to TNFα-caused barrier loss (Zolotarevsky et al., 2002; Wang et al., 2006; Ye and Ma, 2008). Iglesias et al., recently showed ellagic acid to inhibit TNFα-stimulated MLC2 phosphorylation (Iglesias et al., 2020). In contrast, our data show the activation of MLCK/MLC2 in a none-inflammatory state and suggest that MLC2-triggered TJ regulation is not necessarily related to barrier loss as it seems to depend on the specific type of TJ protein affected.

Within the present study, the impact of punicalagin, ellagic acid and urolithin A on inflammation-induced barrier dysfunction was examined in the HT-29/B6 cell culture model, which is a very well-studied inflammation model and more sensitive to TNFα than Caco-2 cells. TNFα is known to up-regulate the expression of sealing claudin-1 and channel forming claudin-2, at which claudin-1 is additionally redistributed from the TJ in HT-29/B6. Together with enhanced epithelial apoptosis, these TJ changes are reported to critically contribute to the TNFα-caused barrier loss (Gitter et al., 2000; Mankertz et al., 2009; Amasheh et al., 2010). In contrast to urolithin A, neither punicalagin nor ellagic acid were effective to inhibit the TNFα-induced TER drop in HT-29/B6. Moreover, urolithin A impeded up-regulation of caudin-1 and -2 and seemed at least partially to prevent redistribution of claudin-1. These effects of urolithin A are very similar to effects we observed in a former study on the ginger-derived pungent component 6-shogaol that also impeded the TNFα-induced up-regulation of claudin-2 and disassembly of claudin-1 (Luettig et al., 2016). Furthermore, TNFα enhances epithelial apoptosis contributing to epithelial leakiness (Gitter et al., 2000). Urolithin A did not inhibit TNFα-caused epithelial apoptosis in HT-29/B6, but even seemed to stimulate it slightly. This is in concordance with other studies that showed ellagitannins from pomegranate and urolithin metabolites to inhibit proliferation and to induce apoptosis in HT-29 cells (Kasimsetty et al., 2010).

These very distinct effects of punicalagin, ellagic acid and urolithin A on intestinal barrier function in the two cell models seem rational, because the bioavailability of these compounds differs along the intestine. Punicalagin was reported to be hydrolyzed already during the stomach passage where it yields ellagic acid. Punicalagin itself probably does not reach the intestine in high amounts, while ellagic acid might predominantly interact with the enterocytes of the ileum. As reported from clinical trials, ellagic acid was not detected in high amounts in the colonic mucosa (Nuñez-Sánchez et al., 2014). In contrast, an increasing gradient of urolithins from the jejunum to the distal colon was described in an animal study (Espín et al., 2007). So far, only very few studies addressed the question, how much punicalagin has to be ingested to reach effective intestinal concentrations of ellagic acid or urolithin A. In the present study, the effects seemed to depend on the optimal dosage, which was figured out for each compound. González-Sarrías et al. simulated gastrointestinal digestion of pomegranate extracts yielding around 500 µM ellagic acid, while plasma concentrations remained low at 100  nM (Nuñez-Sánchez et al., 2014). Subsequently, there are more studies needed to elucidate dosage, intestinal side of conversion, and bioavailability of these components *in vivo*.

## Conclusion

Our study reveals that the punicalagin metabolites ellagic acid and urolithin A have a protective impact on barrier function *in vitro*. These findings support the hypothesis that therapeutically application might act preventive by strengthening and protecting the epithelial barrier in case of diarrhea or inflammation. Moreover, the characterization of these compounds might be of interest for the development of multimodal functional food in the future.

## Data Availability

The raw data supporting the conclusions of this article will be made available by the authors, without undue reservation.

## Abbreviations

DAPI, 4′,6-Diamidin-2-phenylindol; EA, ellagic acid; ERK1/2; extracellular signal-regulated kinases 1/2; LSM, laser scanning microscopy; MLC2, Myosin Light Chain two; MLCK, myosin light chain kinase; NFκB, nuclear factor 'kappa-light-chain-enhancer' of activated B-cells; P, permeability; PCl, chloride permeability; PNa, sodium permeability; Puni, punicalagin; STAT3, signal transducers and activator of transcription 3; TER, transepithelial resistance; TJ, tight junction; TJs, tight junctions; TNFα, tumor necrosis factor α; UroA, urolithin A; ZO, zonula occludens protein.
